# Supplementary material for: 11β-HSD1 inhibition in men mitigates prednisolone-induced adverse effects in a proof-of-concept randomised double-blind placebo-controlled trial
Source: Nat Commun. 2023 Feb 23;14:1025. doi: 10.1038/s41467-023-36541-w (PMC9950480; doi:10.1038/s41467-023-36541-w)
Supplement: Supplementary file 1 — Supplementary Information [file 41467_2023_36541_MOESM1_ESM.pdf]

Supplementary information

Supplementary notes

AZD4017

Efficacy and Dose selection:

AZD4017 is a competitive, fully reversible inhibitor of human recombinant 11β-HSD1 (IC_{50} 2nM) and of 11β-HSD1 activity in isolated human adipocytes (1.8nM) with cortisone as substrate. It is selective (>2000x) over human recombinant 11β-HSD2 and other closely homologous enzymes *in vitro*. AZD4017 has limited activity in pre-clinical species other than the cynomolgus monkey. However, related tool compounds with activity in all species are available; e.g. in diet-induced obese mice, a rodent-active AZ 11β-HSD1 inhibitor induced significant, and approximately half-maximal, reduction in adipose mass and weight gain when compound exposure was ~1 x IC_{50}.

In single ascending dose (SAD) studies, Caucasian and Japanese volunteers were exposed to AZD4017 dosed up to 750mg BID. In a multiple ascending dose (MAD) study, volunteers received single doses of AZD4017 followed by repeated doses ranging from 75mg QD up to 900mg BID for 9 days. Activation of the hypothalamo-pituitary-adrenal (HPA) axis was demonstrated by an increase in ACTH and DHEAs levels and by an increase of total urinary glucocorticoid metabolites. However, serum cortisol and testosterone levels were unchanged.

In a 9-day proof-of-mechanism (PoM) study, AZD4017 (1200mg QD) significantly inhibited hepatic 11β-HSD1 activity as measured by an oral prednisone challenge (prednisone to prednisolone conversion). Measurements of urine glucocorticoid metabolites further indicated an inhibitory effect on whole body 11β-HSD1 activity. Specifically within adipose tissue, a MAD and PoM study in abdominally obese subjects demonstrated inhibition of 11β-HSD1 after single dosing, yet no sustained inhibitory effect in adipose tissue after repeated doses at the tested dose levels. However, *ex vivo* investigations suggest the possibility to obtain an inhibition after repeated dosing at high AZD4017

concentrations[1]. In a 28-day, phase 2a study in patients with raised intraocular pressure, AZD4017 dosed at 400mg BID, produced plasma concentrations of ~10 x IC_{50}. In two externally sponsored clinical studies, a regimen of AZD4017 400mg BID administered orally for 12 weeks led to marked and significant inhibition of systemic 11BHSD1[2,3].

Prednisolone

Dose selection:

The dose of prednisolone was carefully considered and selected based upon the need to demonstrate adverse effects using the chosen sensitive methodologies, balanced against the avoidance of harm (such as adrenal suppression) in a healthy volunteer cohort. Published data suggests that adrenal suppression is unlikely after one week of administration, but can occur after longer durations of administration[4] and therefore a duration of one week was selected. In addition, work from our group and others has suggested that doses of prednisolone of 10mg or less do not have a significant metabolic impact [5-8]. We therefore selected a dose of 20mg prednisolone for one week where there is evidence of adverse metabolic outcomes and is a dose that is clinically relevant and frequently prescribed. The aim of the study was therefore to ameliorate some (or all) of these outcomes through co-administration with AZD4017.

Supplementary figure 1: Detailed study protocol schematic to trial the impact of selective 11β-hydroxysteroid dehydrogenase type 1 inhibition with AZD4017 to limit the adverse effects of prednisolone.

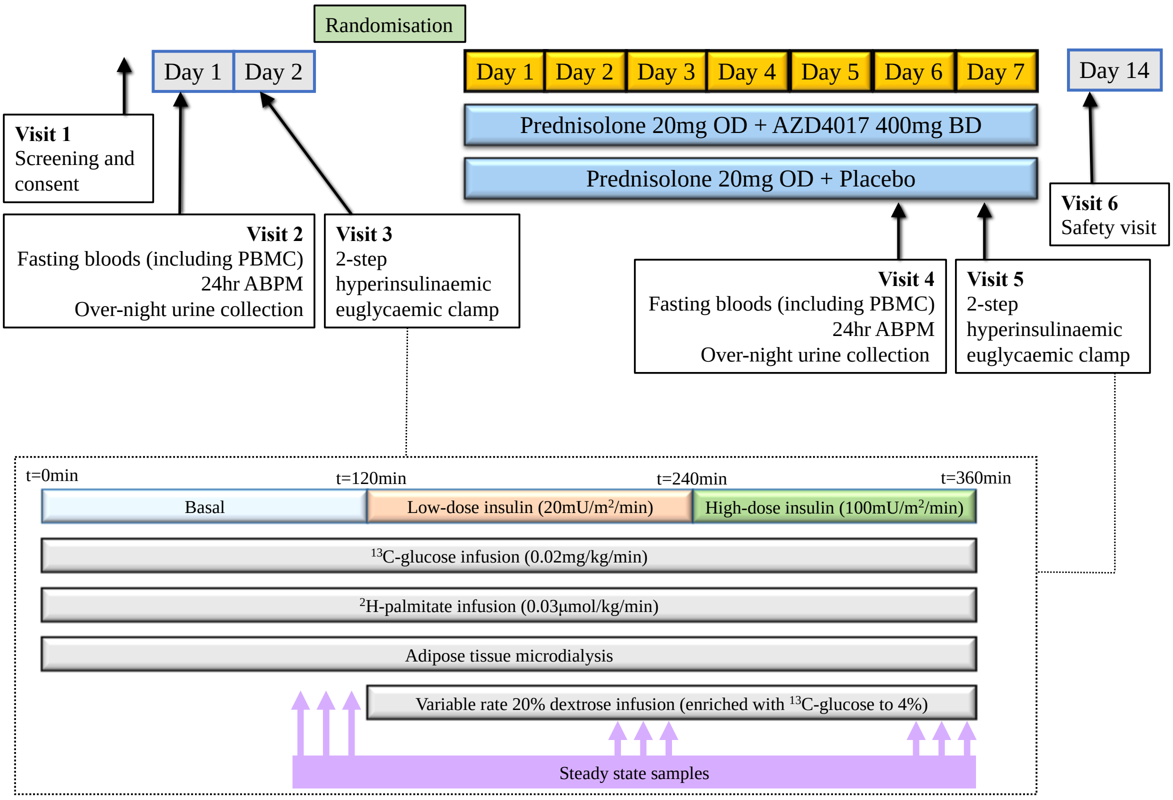

Supplementary Table 1. Primary and secondary outcomes following 7 days treatment with prednisolone (20mg once-daily) and either AZD4017 or placebo. Data are mean (SD) or median (Q1 to Q3). 15 participants from the AZD4017+prednisolone group and 15 from the placebo+prednisolone group were included in the analysis unless otherwise specified. CTX=C-terminal telopeptide. EGP=endogenous glucose production. NEFA=non-esterified fatty acids. P1NP=procollagen type 1 N-terminal propeptide. TAG=triglycerides. VZV=Varicella zoster virus.

† n=14 in AZD4017 group, ‡ n=14 in the placebo group, § n=12 in the AZD4017 group and n=13 in the placebo group, # n=11 in the AZD4017 group and n=13 in the placebo group, ¶ n=12 in the AZD4017 group and n=12 in the placebo group, * n=11 in the AZD4017 group and n=12 in the placebo group, ** n=14 in the AZD4017 group and n=14 in the placebo group, ∯ n=13 in the AZD4017 group and n=14 in the placebo group. Between group statistical analysis was performed using generalised linear models adjusting for baseline variability in each specified outcome. Within subject changes were determined using paired t-tests or Wilcoxon signed-rank test according to the distribution of data.

	**AZD4017 + prednisolone (n=15)**	**Placebo + prednisolone (n=15)**	**Between group p-value**
**Glucose disposal (Low insulin) (GdL) (mg/kg•min)†**			
Baseline	4.59 (1.98)	4.61 (2.28)	
7 days post treatment	4.00 (2.65)	3.06 (1.48)	
Change over 7 days	-0.81 (-1.91 to -0.28)	-1.35 (-2.43 to -0.26)	0.17
Difference within group (p value)	0.32	0.009	
**Glucose disposal (High insulin) (GdH) (mg/kg•min)**			
Baseline	13.13 (3.67)	12.72 (3.73)	
7 days post treatment	10.97 (3.26)	9.83 (2.33)	
Change over 7 days	-2.16 (2.89)	-2.88 (2.91)	0.26

Difference within group (p value)	0.012	0.0018	
**M/I value (Low insulin) (mg/kg•min per mU/mL)**			
Baseline	28.11 (16.58 to 37.00)	30.98 (18.72 to 35.45)	
7 days post treatment	17.19 (6.83 to 28.01)	14.41 (9.56 to 18.04)	
Change over 7 days	-2.67 (17.48)	-10.60 (10.33)	0.15
Difference within group (p value)	0.63	0.0014	
**M/I value (High insulin) (mg/kg•min per mU/mL)**			
Baseline	13.26 (10.01 to 15.20)	15.44 (11.07 to 18.66)	
7 days post treatment	14.71 (8.84 to 16.68)	9.87 (7.41 to 11.99)	
Change over 7 days	-0.51 (-3.76 to 2.53)	-3.72 (-7.25 to -0.98)	0.17
Difference within group (p value)	0.71	0.0049	
**Ra Glucose (mg/kg•min)**			
Baseline	1.34 (1.23 to 1.47)	1.35 (1.26 to 1.44)	
7 days post treatment	1.44 (1.34 to 1.47)	1.42 (1.35 to 1.47)	
Change over 7 days	0.08 (-0.02 to 0.12)	0.06 (-0.02 to 0.13)	0.55
Difference within group (p value)	0.048	0.051	
**EGP (mg/kg•min)†**			
Baseline	0.66 (0.61 to 0.78)	0.68 (0.55 to 0.87)	
7 days post treatment	0.81 (0.71 to 0.94)	1.00 (0.97 to 1.17)	
Change over 7 days	0.16 (-0.03 to 0.29)	0.35 (0.12 to 0.49)	0.11
Difference within group (p value)	0.016	0.013	
**Fasting glucose (mmol/L)**			

Baseline	4.6 (4.4 to 5.4)	4.9 (4.5 to 5.1)	
7 days post treatment	4.9 (4.7 to 5.6)	4.9 (4.8 to 5.1)	
Change over 7 days	0.3 (0.3)	0.1 (0.5)	0.24
Difference within group (p value)	0.0095	0.37	
**Fasting Insulin (mU/L)**			
Baseline	3.13 (1.83 to 6.18)	2.84 (1.44 to 4.88)	
7 days post treatment	2.28 (1.23 to 5.32)	3.77 (2.20 to 5.01)	
Change over 7 days	-0.20 (2.94)	0.48 (1.99)	0.63
Difference within group (p value)	0.89	0.36	
**TAG (Basal) (µmol/L)**			
Baseline	370.2 (339.2 to 610.0)	365.1 (302.5 to 597.9)	
7 days post treatment	374.0 (313.7 to 493.0)	590.9 (526.3 to 879.9)	
Change over 7 days	-50.0 (198.4)	258.2 (240.7)	0.0010
Difference within group (p value)	0.35	0.0004	
**TAG (Low insulin) (µmol/L)**			
Baseline	306.2 (250.8 to 539.8)	260.3 (213.7 to 477.8)	
7 days post treatment	259.2 (172.4 to 362.2)	386.1 (281.4 to 534.9)	
Change over 7 days	-46.9 (-134.9 to 3.5)	125.8 (18.0 to 155.9)	0.0014
Difference within group (p value)	0.026	0.018	
**TAG (High insulin) (µmol/L)**			
Baseline	233.3 (188.5 to 448.9)	179.5 (157.9 to 428.1)	
7 days post treatment	182.4 (151.9 to 240.7)	248.9 (204.5 to 380.7)	
Change over 7 days	-40.7 (-190.5 to 2.4)	58.1 (-6.9 to 90.2)	0.0069

Difference within group (p value)	0.026	0.15	
**Glycerol (Basal) (µmol/L)**			
Baseline	22.6 (16.2 to 26.8)	20.0 (17.8 to 24.8)	
7 days post treatment	25.8 (20.1 to 31.5)	27.2 (20.0 to 35.2)	
Change over 7 days	3.3 (10.3)	8.3 (9.9)	0.39
Difference within group (p value)	0.36	0.0056	
**Glycerol (Low insulin) (µmol/L)**			
Baseline	8.7 (5.0)	7.6 (3.0)	
7 days post treatment	10.5 (6.3)	12.3 (3.5)	
Change over 7 days	1.5 (0.4 to 2.7)	4.1 (3.3 to 6.0)	0.11
Difference within group (p value)	0.21	<0.0001	
**Glycerol (High insulin) (µmol/L)‡**			
Baseline	5.8 (4.6 to 10.9)	6.8 (4.7 to 8.6)	
7 days post treatment	6.3 (5.2 to 11.2)	9.5 (8.4 to 14.6)	
Change over 7 days	0.2 (-1.2 to 1.1)	2.8 (1.9 to 4.9)	0.089
Difference within group (p value)	0.93	0.00016	
**Adipose interstitial fluid - Glycerol (Basal) (µmol/L•hr)§**			
Baseline	219.5 (171.6 to 281.4)	250.3 (205.0 to 266.8)	
7 days post treatment	266.1 (222.2 to 307.1)	265.0 (202.8 to 316.8)	
Change over 7 days	47.8 (-9.8 to 77.9)	30.3 (-60.0 to 101.5)	0.30
Difference within group (p value)	0.38	0.47	
**Adipose interstitial fluid - Glycerol (Low insulin) (µmol/L•hr)#**			

Baseline	132.3 (85.1 to 163.5)	114.0 (87.3 to 142.3)	
7 days post treatment	178.8 (79.0 to 272.8)	165.5 (138.0 to 228.0)	
Change over 7 days	36.6 (71)	87.3 (109.3)	0.38
Difference within group (p value)	0.28	0.011	
**Adipose interstitial fluid - Glycerol (High insulin) (µmol/L•hr)¶**			
Baseline	84.6 (16.8)	72.4 (8.5)	
7 days post treatment	110.5 (19.7)	141.6 (15.1)	
Change over 7 days	-3.9 (-15.4 to 46.8)	71.8 (28.1 to 109.3)	0.16
Difference within group (p value)	0.22	0.0013	
**NEFA (Basal) (µmol/L)**			
Baseline	309.9 (257.9 to 423.1)	278.9 (243.2 to 433.1)	
7 days post treatment	452.1 (266.9 to 667.8)	452.7 (294.9 to 629.9)	
Change over 7 days	-4.6 (-93.4 to 241.2)	129.2 (-5.1 to 196.9)	0.72
Difference within group (p value)	0.36	0.073	
**NEFA (Low insulin) (µmol/L)**			
Baseline	71.9 (54.7 to 125.3)	83.7 (52.7 to 141.3)	
7 days post treatment	155 (62.5 to 216.8)	129.3 (98.2 to 299.6)	
Change over 7 days	2.4 (-4.1 to 69.5)	33.2 (21.3 to 71.4)	0.25
Difference within group (p value)	0.36	0.073	
**NEFA (High insulin) (µmol/L)**			
Baseline	57.9 (35.4 to 135.1)	54.3 (42.3 to 139.2)	
7 days post treatment	57.2 (38.2 to 95.8)	68.3 (46.3 to 218.6)	
Change over 7 days	1.2 (-9.5 to 43.4)	16.3 (2.1 to 55.8)	0.66

Difference within group (p value)	0.60	0.022	
**Ra Palmitate (Basal) (mg/kg•min)***			
Baseline	1.90 (0.46)	1.93 (0.69)	
7 days post treatment	1.99 (0.78)	1.82 (0.59)	
Change over 7 days	0.10 (0.86)	-0.12 (0.69)	0.51
Difference within group (p value)	0.71	0.57	
**Ra Palmitate (Low insulin) (mg/kg•min)***			
Baseline	0.66 (0.56 to 0.94)	0.67 (0.47 to 0.98)	
7 days post treatment	0.81 (0.69 to 1.16)	0.81 (0.71 to 0.95)	
Change over 7 days	0.10 (-0.05 to 0.41)	0.16 (-0.15 to 0.32)	0.62
Difference within group (p value)	0.32	0.52	
**Ra Palmitate (High insulin) (mg/kg•min)***			
Baseline	0.77 (0.68 to 0.97)	0.67 (0.50 to 1.06)	
7 days post treatment	0.60 (0.51 to 0.86)	0.64 (0.55 to 0.82)	
Change over 7 days	-0.17 (-0.44 to -0.03)	-0.03 (-0.22 to 0.15)	0.31
Difference within group (p value)	0.024	0.79	
**Daytime blood pressure – Systolic (mm Hg)****			
Baseline	125.0 (6.3)	127.8 (8.8)	
7 days post treatment	127.4 (8.8)	130.3 (9.8)	
Change over 7 days	2.4 (7.0)	2.5 (6.1)	0.66
Difference within group (p value)	0.22	0.15	
**Daytime blood pressure – Diastolic (mm Hg)****			

Baseline	74.6 (7.2)	79.1 (7.4)	
7 days post treatment	75.9 (7.4)	79.4 (6.8)	
Change over 7 days	1.4 (6.2)	0.3 (5.8)	0.92
Difference within group (p value)	0.42	0.86	
**Nighttime blood pressure – Systolic (mm Hg) ∮**			
Baseline	111.1 (7.7)	111.8 (9.0)	
7 days post treatment	112.1 (9.9)	116.4 (13.6)	
Change over 7 days	1.0 (11.5)	4.6 (10.6)	0.19
Difference within group (p value)	0.76	0.13	
**Nighttime blood pressure – Diastolic (mm Hg) ∮**			
Baseline	63.0 (61.0 to 64.0)	63.5 (60.5 to 71.0)	
7 days post treatment	65.0 (55.0 to 69.0)	68.5 (65.8 to 74.5)	
Change over 7 days	0.7 (8.1)	4.6 (8.6)	0.03
Difference within group (p value)	0.72	0.064	
**Osteocalcin (ng/mL)**			
Baseline	7.79 (6.18 to 11.69)	9.12 (7.44 to 9.76)	
7 days post treatment	8.13 (6.80 to 10.31)	4.16 (3.46 to 5.04)	
Change over 7 days	0.00 (-3.46 to 2.18)	-3.86 (-5.78 to -2.74)	<0.0001
Difference within group (p value)	0.64	<0.0001	
**P1NP (ng/mL)**			
Baseline	54.1 (44.2 to 66.6)	52.8 (39.8 to 63.1)	
7 days post treatment	48.9 (44.0 to 65.6)	40.3 (33.4 to 46.9)	
Change over 7 days	-2.2 (-5.4 to 1.8)	-7.2 (-11.2 to -4.8)	0.0014

Difference within group (p value)	0.073	0.0001	
**CTX (ng/mL)**			
Baseline	0.51 (0.34 to 0.67)	0.44 (0.27 to 0.48)	
7 days post treatment	0.55 (0.37 to 0.71)	0.43 (0.35 to 0.59)	
Change over 7 days	0.04 (-0.06 to 0.08)	0.06 (0.003 to 0.14)	0.094
Difference within group (p value)	0.38	0.0028	
**VZV OX40 (% of CD25+CD134+)**			
Baseline	1.08 (0.26 to 1.02)	1.41 (0.31 to 1.20)	
7 days post treatment	0.57 (0.13 to 0.50)	0.57 (0.17 to 0.64)	
Change over 7 days	-0.40 (-0.55 to -0.10)	-0.30 (-1.85 to 0.00)	0.81
Difference within group (p value)	0.016	0.011	
**PHA OX40 (% of CD25+CD134+)**			
Baseline	59.4 (51.6 to 74.4)	63.2 (56.3 to 75.0)	
7 days post treatment	54.4 (41.2 to 63.2)	40.3 (27.7 to 55.8)	
Change over 7 days	-10.2 (14.4)	-21.7 (14.0)	0.046
Difference within group (p value)	0.018	0.0004	

Supplementary table 2. Targeted serum proteomic analysis in patients treated with placebo+prednisolone *vs.* AZD4017+prednisolone. Pre- and post-treatment cytokine concentrations and absolute differences are presented for each arm. Values are reported as mean and SD. P values <0.05 are highlighted in bold. ([€]generalized linear model (GLM) p-value after adjusting for the pre-treatment values. [¥]Wilcoxon sign rank test p-value [ǂ]Non-parametric GLM p-value after adjusting for the pre-treatment values)

	AZD4017 + prednisolone (n=15)				Prednisolone + placebo (n=15)				
**Cytokine**	**Pre-treatment (pg/ml)**	**Post-treatment (pg/ml)**	**p value**	**Δ Pre- vs. Post- (pg/ml)**	**Pre-treatment (pg/ml)**	**Post-treatment (pg/ml)**	**P value**	**Δ Pre- vs. Post- (pg/ml)**	**P value (Δ AZD4017 vs. Δ placebo)**
**CCL8**	30.50 (11.61)	26.31 (11.36)	0.210	-4.19 (12.35)	35.20 (6.07)	30.36 (8.77)	**0.005**	-4.84 (5.66)	0.854
**IL33**	0.12 (0.08)	0.09 (0.04)	0.082	-0.04 (0.08)	0.17 (0.10)	0.12 (0.08)	**0.033**	-0.05 (0.08)	0.838
**CXCL12**	131.33 (21.81)	114.29 (26.00)	**0.035**	-17.04 (28.2)	169.27 (57.57)	142.74 (71.10)	**0.001**	-26.52 (23.36)	0.325
**OLR1**	24.06 (10.00)	22.24 (16.68)	0.723	-1.81 (19.45)	24.49 (13.49)	20.51 (14.05)	0.422	-3.98 (18.62)	0.758
**IL27**	7.09 (4.29)	5.60 (3.45)	0.163	-1.49 (3.93)	7.64 (5.34)	9.14 (7.73)	0.173	1.5 (4.05)	**0.049**
**IL2**	0.01 (0.01)	0.01 (0.01)	0.515	0 (0.01)	0.01 (0.01)	0.01 (0.01)	0.164	0 (0.01)	0.571
**CXCL9**	92.25 (103.43)	57.13 (27.96)	0.183	-35.12 (97.08)	108.80 (149.08)	59.36 (52.76)	0.079	-49.44 (101.23)	0.695
**TGFA**	5.88 (1.82)	5.02 (2.10)	0.109	-0.87 (1.96)	5.48 (0.91)	5.44 (1.17)	0.876	-0.05 (1.18)	0.177
**IL1B**	0.06 (0.03)	0.06 (0.06)	0.879	0 (0.04)	0.06 (0.06)	0.23 (0.73)	0.377	0.17 (0.73)	0.392
**IL6**	3.33 (2.99)	2.40 (1.28)	0.150	-0.93 (2.36)	2.24 (1.17)	2.54 (1.55)	0.498	0.3 (1.65)	0.110
**IL4**	0.02 (0.00)	0.02 (0.00)	0.899	0 (0)	0.02 (0.00)	0.02 (0.00)	0.687	0 (0)	0.877
**TNFSF12**	399.19 (87.04)	348.00 (81.97)	0.083	-51.19 (106.32)	405.10 (72.04)	332.71 (77.16)	**0.004**	-72.39 (80.31)	0.543
**TSLP**	0.20 (0.19)	0.11 (0.13)	**0.000**	-0.11 (0.06)	0.47 (1.17)	0.40 (0.99)	0.216	-0.09 (0.24)	0.783
**CCL11**	349.23 (102.96)	321.64 (94.45)	0.111	-27.59 (62.87)	370.48 (103.16)	371.89 (106.82)	0.931	1.4 (61.64)	0.213
**HGF**	108.75 (19.49)	104.03 (23.38)	0.508	-4.72 (26.9)	117.40 (19.04)	103.70 (22.14)	**0.004**	-13.7 (15.41)	0.271
**FLT3LG**	97.70 (30.67)	89.33 (35.51)	0.332	-8.37 (32.25)	91.62 (24.73)	66.25 (17.96)	**0.000**	-25.37 (18.76)	0.089
**IL17F**	0.58 (0.76)	0.36 (0.35)	0.100	-0.22 (0.48)	1.20 (1.86)	1.01 (1.87)	**0.008**	-0.19 (0.24)	0.829
**IL7**	0.55 (0.15)	0.58 (0.21)	0.463	0.03 (0.15)	0.73 (0.60)	0.64 (0.57)	0.082	-0.09 (0.19)	0.064
**IL13**	1.77 (4.43)	1.64 (2.33)	0.668	-0.44 (3.28)	17.48 (56.87)	17.97 (59.04)	0.092	-0.75 (1.55)	0.753
**IL18**	269.09 (88.23)	234.84 (100.03)	0.237	-34.25 (107.44)	255.19 (94.03)	214.11 (90.97)	**0.005**	-41.08 (48.41)	0.824
**CCL13**	492.69 (277.43)	414.49 (309.74)	0.282	-78.2 (270.94)	489.14 (177.08)	373.06 (197.74)	**0.000**	-116.08 (88.71)	0.611
**TNFSF10**	516.38 (119.90)	484.01 (123.96)	0.369	-32.37 (134.98)	491.21 (74.87)	463.72 (74.26)	0.322	-27.5 (103.77)	0.913
**CXCL10**	114.75 (87.67)	79.67 (35.53)	0.076	-35.07 (70.87)	111.67 (70.15)	56.63 (25.09)	**0.001**	-55.04 (52.75)	0.389
**IFNG**	0.19 (0.06)	0.17 (0.08)	0.530	-0.01 (0.08)	0.39 (0.38)	0.17 (0.10)	**0.023**	-0.22 (0.33)	**0.028**
**IL10**	7.21 (3.39)	8.90 (5.47)	0.196	1.7 (4.84)	7.61 (3.17)	7.55 (4.62)	0.928	-0.07 (2.73)	0.230
**CCL19**	121.54 (29.97)	109.61 (60.87)	0.307	-11.93 (43.6)	169.67 (136.78)	100.25 (99.68)	**0.000**	-69.42 (48.54)	**0.002**
**TNF**	19.49 (14.26)	15.49 (8.05)	0.055	-3.99 (7.37)	15.86 (2.67)	12.96 (2.65)	**0.001**	-2.89 (2.84)	0.594
**IL15**	11.17 (2.41)	10.15 (2.65)	0.228	-1.02 (3.12)	11.19 (2.06)	11.95 (2.38)	0.309	0.76 (2.81)	0.112
**CCL3**	5.71 (2.13)	5.18 (2.07)	0.274	-0.53 (1.8)	5.78 (1.74))	5.17 (1.78)	0.099	-0.61 (1.33)	0.895
**CXCL8**	5.09 (1.43)	4.60 (1.47)	0.322	-0.5 (1.87)	5.82 (1.59)	5.06 (2.01)	0.078	-0.76 (1.55)	0.677
**MMP12**	239.47 (87.10)	225.69 (176.90)	0.693	-13.78 (132.35)	246.52 (97.79)	119.10 (37.24)	**0.000**	-127.42 (82.28)	**0.009**
**CSF2**	0.14 (0.06)	0.13 (0.06)	0.325	-0.02 (0.06)	0.22 (0.11)	0.19 (0.13)	0.580	-0.03 (0.18)	0.844
**CSF3**	121.05 (53.60)	100.12 (34.90)	0.068	-20.93 (40.92)	101.77 (37.00)	110.14 (50.76)	0.287	8.37 (29.29)	**0.032**
**VEGFA**	198.66 (35.41)	194.18 (58.21)	0.799	-4.49 (67.09)	204.27 (37.88)	200.57 (53.96)	0.775	-3.7 (49.21)	0.971
**IL17C**	23.46 (17.39)	18.85 (8.20)	0.352	-4.61 (18.57)	19.04 (6.52)	20.51 (7.87)	0.395	1.47 (6.49)	0.241
**EGF**	34.59 (27.19)	39.45 (37.09)	0.633	4.86 (38.53)	36.82 (33.09)	33.10 (22.14)	0.539	-3.72 (22.84)	0.465
**CCL2**	315.25 (65.71)	283.66 (104.92)	0.338	-31.59 (123.22)	329.00 (100.91)	323.11 (94.12)	0.793	-5.89 (85.18)	0.512
**IL17A**	0.56 (0.44)	0.54 (0.65)	0.900	-0.02 (0.73)	0.45 (0.29)	0.29 (0.29)	**0.029**	-0.17 (0.27)	0.486
**OSM**	0.21 (0.10)	0.39 (0.49)	0.187	0.18 (0.5)	0.32 (0.22)	0.37 (0.15)	0.394	0.05 (0.21)	0.355
**CSF1**	118.98 (14.94)	111.43 (22.80)	0.254	-7.55 (24.58)	117.86 (13.92)	108.97 (15.59)	0.066	-8.89 (17.26)	0.864
**CCL4**	77.15 (60.51)	62.16 (31.73)	0.212	-14.99 (44.42)	68.66 (18.96)	62.69 (22.17)	0.242	-5.97 (18.94)	0.475
**CXCL11**	471.14 (385.30)	317.58 (192.49)	0.125	-153.55 (364.81)	520.24 (382.38)	268.81 (138.96)	**0.007**	-251.43 (307.85)	0.434
**LTA**	9.18 (2.29)	7.84 (2.34)	**0.049**	-1.34 (2.41)	9.68 (2.41)	7.10 (1.88)	**0.000**	-2.58 (2.06)	0.141
**CCL7**	2.32 (1.09)	2.02 (0.65)	0.221	-0.3 (0.91)	1.99 (0.56)	1.51 (0.58)	**0.000**	-0.49 (0.39)	0.472
**MMP1**	351.30 (178.17)	440.03 (448.85)	0.363	88.73 (365.11)	490.48 (264.87)	470.54 (284.80)	0.664	-19.94 (173.95)	0.307

Supplementary References:

1. Morentin Gutierrez P, Gyte A, deSchoolmeester J, *et al.* Continuous inhibition of 11β-hydroxysteroid dehydrogenase type I in adipose tissue leads to tachyphylaxis in humans and rats but not in mice. Br J Pharmacol. 2015 Oct;172(20):4806-16
2. Markey K, Mitchell J, Botfield H, *et al.* 11β-Hydroxysteroid dehydrogenase type 1 inhibition in idiopathic intracranial hypertension: a double-blind randomized controlled trial. Brain Commun. 2020;2(1):fcz050. doi: 10.1093/braincomms/fcz050.
3. Ajjan R, Hensor E, Shams K, *et al.* https://www.medrxiv.org/content/10.1101/2021.03.23.21254200v1)
4. Prete A, Bancos I. Glucocorticoid induced adrenal insufficiency. BMJ 2021;374:n1936
5. Jacobsson M, van Raalte DH, Heijboer AC, *et al.* Short-Term Glucocorticoid Treatment Reduces Circulating Sclerostin Concentrations in Healthy Young Men: A Randomized, Placebo-Controlled, Double-Blind Study. JBMR Plus. 2020;4(8):e10341.
6. Othonos N, Marjot T, Woods C, *et al.* Co-administration of 5α-reductase Inhibitors Worsens the Adverse Metabolic Effects of Prescribed Glucocorticoids. J Clin Endocrinol Metab. 2020;105(9):e3316-28
7. Pofi R, Bonaventura I, Othonos N *et al.* Glucocorticoid treatment is associated with dose-dependent effects in healthy male volunteers [abstract]. Endocrine Abstracts. 2019; 65:p242. doi:10.1530/endoabs.65.P242
8. van Raalte DH, Nofrate V, Bunck MC, *et al.* Acute and 2-week exposure to prednisolone impair different aspects of beta-cell function in healthy men. Eur J Endocrinol. 2010;162(4):729-35
