# Supplementary material for: 11β-HSD1 inhibition in men mitigates prednisolone-induced adverse effects in a proof-of-concept randomised double-blind placebo-controlled trial
Source: Nat Commun. 2023 Feb 23;14:1025. doi: 10.1038/s41467-023-36541-w (PMC9950480; doi:10.1038/s41467-023-36541-w)
Supplement: Supplementary file 3 — Reporting Summary [file 41467_2023_36541_MOESM3_ESM.pdf]

nature portfolio

Corresponding author(s): Jeremy Tomlinson

Last updated by author(s): Jan 10, 2023

Reporting Summary

Nature Portfolio wishes to improve the reproducibility of the work that we publish. This form provides structure for consistency and transparency in reporting. For further information on Nature Portfolio policies, see our Editorial Policies and the Editorial Policy Checklist.

Statistics

For all statistical analyses, confirm that the following items are present in the figure legend, table legend, main text, or Methods section.

n/a	Confirmed	
☐	☒	The exact sample size (n) for each experimental group/condition, given as a discrete number and unit of measurement
☐	☒	A statement on whether measurements were taken from distinct samples or whether the same sample was measured repeatedly
☐	☒	The statistical test(s) used AND whether they are one- or two-sided<br>*Only common tests should be described solely by name; describe more complex techniques in the Methods section.*
☐	☒	A description of all covariates tested
☐	☒	A description of any assumptions or corrections, such as tests of normality and adjustment for multiple comparisons
☐	☒	A full description of the statistical parameters including central tendency (e.g. means) or other basic estimates (e.g. regression coefficient) AND variation (e.g. standard deviation) or associated estimates of uncertainty (e.g. confidence intervals)
☐	☒	For null hypothesis testing, the test statistic (e.g. F, t, r) with confidence intervals, effect sizes, degrees of freedom and P value noted<br>*Give P values as exact values whenever suitable.*
☒	☐	For Bayesian analysis, information on the choice of priors and Markov chain Monte Carlo settings
☒	☐	For hierarchical and complex designs, identification of the appropriate level for tests and full reporting of outcomes
☒	☐	Estimates of effect sizes (e.g. Cohen's d, Pearson's r), indicating how they were calculated

Our web collection on statistics for biologists contains articles on many of the points above.

Software and code

Policy information about availability of computer code

Data collection	No software was used for data collection.
Data analysis	Data was analysed using SAS 9.4 for Windows (Copyright © 2013, SAS Institute Inc., Cary, NC, USA) and STATA for Windows, StataCorp. 2021 (Stata Statistical Software: Release 17. College Station, TX: StataCorp LLC)

For manuscripts utilizing custom algorithms or software that are central to the research but not yet described in published literature, software must be made available to editors and reviewers. We strongly encourage code deposition in a community repository (e.g. GitHub). See the Nature Portfolio guidelines for submitting code & software for further information.

Data

Policy information about availability of data

All manuscripts must include a data availability statement. This statement should provide the following information, where applicable:

- Accession codes, unique identifiers, or web links for publicly available datasets
- A description of any restrictions on data availability
- For clinical datasets or third party data, please ensure that the statement adheres to our policy

Source data are provided with this paper.

Human research participants

Policy information about studies involving human research participants and Sex and Gender in Research.

Reporting on sex and gender	Only male volunteers were included in the study.
Population characteristics	Participants were recruited if they met the following inclusion criteria: male, aged 18-60 years with a BMI of 20-30kg/m2. Key exclusion criteria included diabetes mellitus, hypertension, hypercholesterolemia, use of GC therapy within the last 6 months, taking medications known to impact upon GC metabolism, eGFR <60mL/min/1.73m2 and abnormal liver chemistry.
Recruitment	They were recruited from local advertisement and from the Oxford Biobank (reference 08/H0606/107). Only male participants were recruited and none had significant past medical history. All participants were randomized to each intervention arm (double blind) and there we do not believe that selection bias would have influenced the data.
Ethics oversight	East of England Cambridge East Research Ethics Committee (reference 16/EE/0550).

Note that full information on the approval of the study protocol must also be provided in the manuscript.

Field-specific reporting

Please select the one below that is the best fit for your research. If you are not sure, read the appropriate sections before making your selection.

[x] Life sciences [ ] Behavioural & social sciences [ ] Ecological, evolutionary & environmental sciences

For a reference copy of the document with all sections, see nature.com/documents/nr-reporting-summary-flat.pdf

Life sciences study design

All studies must disclose on these points even when the disclosure is negative.

Sample size	The sample size required to detect a 20% Gd reduction with 80% power and a type I error of 0.05 was calculated to be 13 per group. Allowing for a potential dropout rate of 20%, 32 was the recruitment target for the study.
Data exclusions	Two participants were excluded from the data analysis. One participant from the AZD4017 group was excluded due to <85% compliance with study medication and one participant from the placebo-treated group was excluded for repeated failure to fast before study procedures.
Replication	The study was adequately powered with 15 participants per group and that assays on samples were analysed in duplicates or triplicates.
Randomization	Participants were randomized 1:1 to treatment with AZD4017 (400mg PO twice-daily) or matching placebo, in addition to oral prednisolone (20mg once-daily), according to an independently developed randomisation table, using blocks of four. Participants were randomized sequentially according to their recruitment date.
Blinding	This is a randomised, double-blind, placebo-controlled study.

Reporting for specific materials, systems and methods

We require information from authors about some types of materials, experimental systems and methods used in many studies. Here, indicate whether each material, system or method listed is relevant to your study. If you are not sure if a list item applies to your research, read the appropriate section before selecting a response.

Materials & experimental systems

n/a	Involved in the study	
[x]	[ ]	Antibodies
[x]	[ ]	Eukaryotic cell lines
[x]	[ ]	Palaeontology and archaeology
[x]	[ ]	Animals and other organisms
[ ]	[x]	Clinical data
[x]	[ ]	Dual use research of concern

Methods

n/a	Involved in the study	
[x]	[ ]	ChIP-seq
[x]	[ ]	Flow cytometry
[x]	[ ]	MRI-based neuroimaging

Clinical data

Policy information about clinical studies

All manuscripts should comply with the ICMJE guidelines for publication of clinical research and a completed CONSORT checklist must be included with all submissions.

Clinical trial registration	NCT03111810
Study protocol	https://clinicaltrials.gov/ct2/show/NCT03111810
Data collection	42 participants were assessed for study eligibility between 25th May 2017 and 13th February 2019; 10 failed the screening process leaving 32 participants who were enrolled and randomized. The data was collected at the Clinical Research Unit (CRU), Churchill Hospital (Oxford, UK). The data was collected between 25th May 2017 and 13th February 2019 at the clinical research unit.
Outcomes	The primary endpoint was the change observed in glucose disposal (Gd) from pre-treatment measurement to post-treatment assessment, as measured during a hyperinsulinaemic clamp. Pre-defined secondary endpoints included changes in EGP, lipolysis (systemic and adipose tissue), 24h BP measurements, circulating lipid profiles, osteocalcin, urinary steroid metabolites and immunoinflammatory response as measured by the OX40 assay.
